# Validation and interpretation of machine-learning models for rapid identification of active tuberculosis infection using routine laboratory indicators

**DOI:** 10.3389/fcimb.2025.1718614

**Published:** 2025-12-18

**Authors:** Zhan-Zhong Liu, Quan Yuan, Yu-Dong Zhang, Xue-Di Zhang, Jian Liu, Jia-Wei Yan, Kang-Peng Du, Hui-Jin Chen, Liang Wang

**Affiliations:** 1 th Xuzhou Hospital, Beijing Ditan Hospital Affiliated to Capital Medical University, Xuzhou Infectious Diseases Hospital (The 7^th^ People’s Hospital of Xuzhou), Xuzhou, Jiangsu, China; 2 Department of Laboratory Medicine, Shengli Oilfield Central Hospital, Dongying, Shandong, China; 3 School of Medical Informatics and Engineering, Xuzhou Medical University, Xuzhou, Jiangsu, China; 4 School of 1^st^ Clinical Medicine, Xuzhou Medical University, Xuzhou, Jiangsu, China; 5 Department of Pharmacy, The 6^th^ People’s Hospital of Xuzhou, Xuzhou, Jiangsu, China; 6 Laboratory Medicine, Guangdong Provincial People’s Hospital (Guangdong Academy of Medical Sciences), Southern Medical University, Guangzhou, Guangdong, China

**Keywords:** biochemical test, blood test, machine learning algorithm, *Mycobacterium tuberculosis*, predictive model, routine laboratory indicators

## Abstract

**Introduction:**

Diagnosis of active *Mycobacterium tuberculosis* (Mtb) infection relies on clinical symptoms, imaging, and molecular testing, but these methods are often costly and slow. Consequently, there is an urgent need for a rapid and accessible diagnostic approach that can support early detection and reduce ongoing *tuberculosis* transmission.

**Methods:**

A discovery cohort of 3,829 individuals and an external validation cohort of 405 individuals were included. Six supervised machine learning models were trained using routine laboratory data, and model interpretability was assessed with SHapley Additive exPlanations (SHAP).

**Results:**

Among the six models, XGBoost demonstrated the best diagnostic performance in the internal cohort (accuracy 97.49%; sensitivity 97.56%; specificity 97.42%) and maintained strong performance in the external cohort (accuracy 93.67%; sensitivity 91.56%; specificity 91.13%). SHAP analysis indicated that key predictors reflected characteristic host-response patterns, including inflammation-related hypoalbuminemia, lipid metabolism suppression (HDL-C and LDL-C), altered platelet activity (MPV), and lymphocyte reduction (LYM).

**Conclusion:**

The study presents a high-performing and interpretable machine learning model capable of accurately identifying active Mtb infection using routine blood tests. This low-cost and non-invasive approach has strong potential for application in resource-limited and high-burden settings.

## Graphical Abstract

**Figure:**
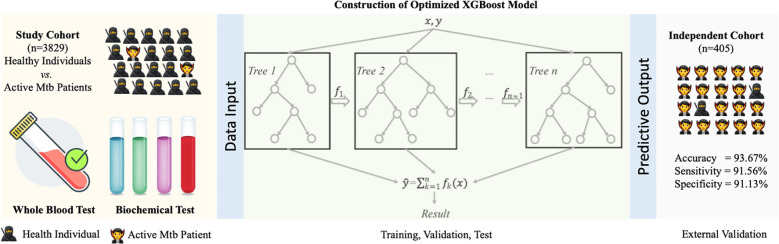

## 1 Background

Tuberculosis (TB), caused by *Mycobacterium tuberculosis* (Mtb), remains a major global health threat, with the World Health Organization (WHO) estimating that there will be approximately 10.6 million new cases and more than 1.6 million deaths worldwide in 2022 (Chakaya et al., 2021; Bagcchi, 2023). Active Mtb infection is the transmissible form of tuberculosis and a major driver of continued spread (Babalola, 2015; Xin et al., 2020). Timely and accurate identification of active Mtb is essential not only for initiating effective treatment but also for interrupting transmission chains and reducing the public health burden (Organization, 2020).

Currently, the diagnosis of active Mtb is based on clinical evaluation, radiographic imaging, and microbiological testing. Conventional methods such as sputum smear microscopy (Desikan, 2013), mycobacterial culture (Azadi et al., 2018), and nucleic acid amplification tests (NAATs), including the Xpert MTB/RIF assay (Kay et al., 2020), have their respective strengths and limitations. Smear microscopy, while widely used, has low sensitivity, particularly in paucibacillary or extrapulmonary TB (Caulfield and Wengenack, 2016). Culture remains the reference standard but requires weeks to yield results (Forbes et al., 2018). Although NAATs provide rapid and sensitive detection, their cost and equipment requirements restrict their use in many low-resource settings (Dinnes et al., 2007). Therefore, there is an urgent need to develop rapid, accurate, and low-cost diagnostic strategies based on existing platform data to improve early detection of active Mtb and reduce ongoing transmission.

In recent years, machine learning (ML) has emerged as a promising approach to extract diagnostic values from high-dimensional clinical data, including routine laboratory indicators (Rabbani et al., 2022; Wan et al., 2025). Routine tests such as complete blood count and biochemical profiles are widely available, inexpensive, and already integrated into standard hospital workflows (Santos-Silva et al., 2024). Multiple studies have demonstrated that ML applied to these indicators can aid disease diagnosis (Kistenev et al., 2022; Liu et al., 2024). For example, Luo et al. developed a gradient boosting model based on routine hematological and biochemical indicators to differentiate active Mtb from latent TB infection (LTBI), achieving high diagnostic performance with sensitivity and specificity exceeding 84% and 91%, respectively, in both internal and external validation cohorts (Luo et al., 2022). Similarly, Dai et al. applied random forest and gradient boosting classifiers to a combination of clinical laboratory indicators to distinguish COVID-19 from community-acquired pneumonia, achieving excellent predictive accuracy (AUROC > 0.85, recall > 0.90), even with a limited number of features (Dai et al., 2021). Therefore, leveraging routine laboratory indicators in combination with machine learning algorithms holds great promise for assisting in the diagnosis of active Mtb infection and other diseases, offering a practical and scalable solution for early disease identification.

In this study, we developed and validated a machine-learning diagnostic model that leverages routinely collected hematological and biochemical laboratory parameters to identify active Mtb infection. To ensure clinical relevance and robustness, we constructed a clinical cohort and systematically compared the performance of multiple machine learning algorithms. Feature importance was subsequently quantified with SHapley Additive exPlanations (SHAP), enabling refined feature selection and enhanced model interpretability. External validation in an independent cohort demonstrated strong generalizability. It highlighted the model’s clinical potential, underscoring that standard laboratory metrics can serve as a powerful adjunctive tool for the diagnosis of active tuberculosis.

## 2 Materials and methods

### 2.1 Study design

**Figure 1:**
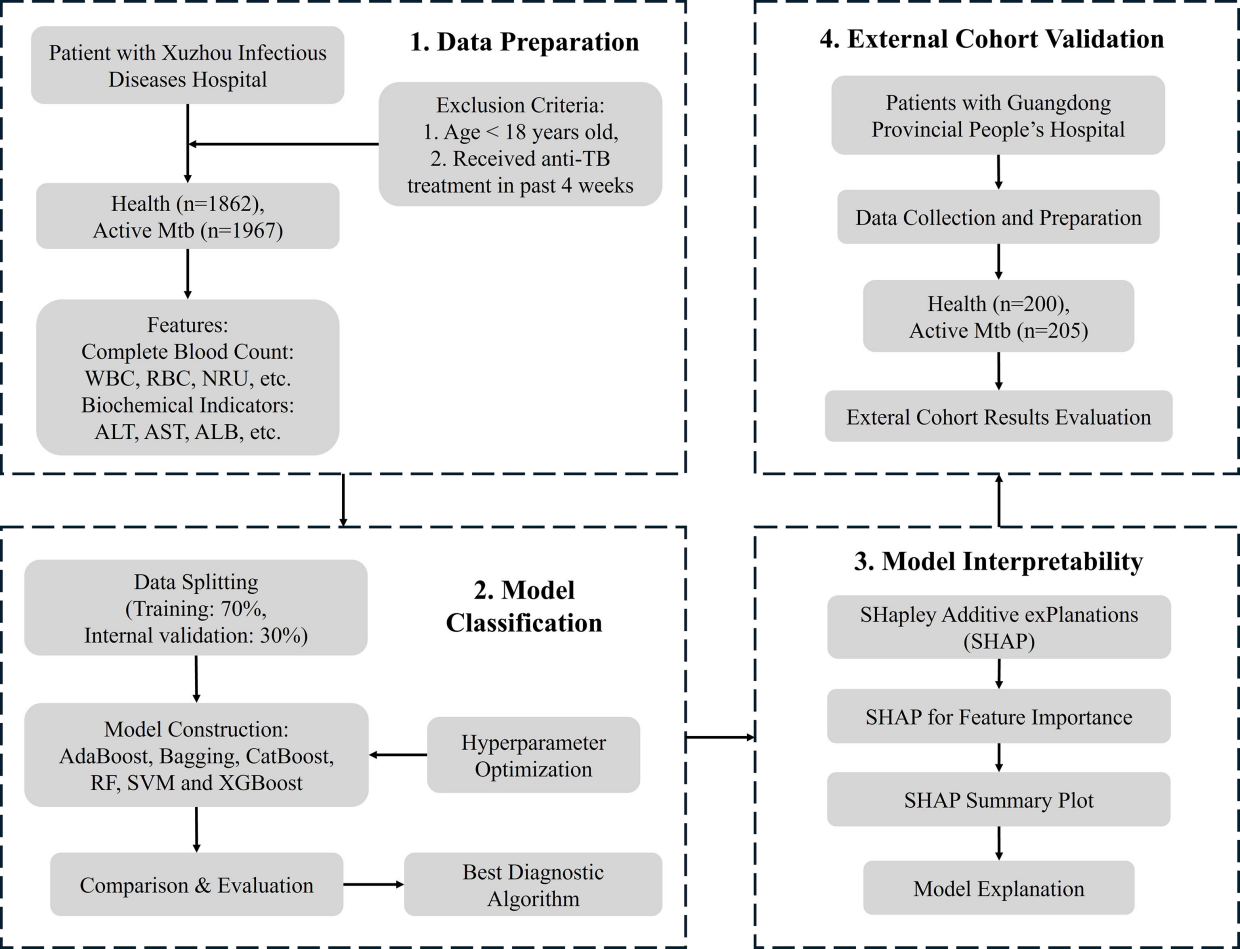
Schematic workflow of the study design: 1. data preparation from clinical and biochemical indicators; 2. model construction and internal validation using multiple machine learning algorithms; 3. model interpretation with SHAP analysis; 4. external cohort validation in an independent hospital.

The present study was conducted at Xuzhou Infectious Diseases Hospital (The 7^th^ Hospital of Xuzhou). Biochemical and hematological data were retrospectively collected from 3,829 in-patients admitted between January 2016 and December 2022, constituting the discovery cohort. An independent validation cohort comprised 405 participants recruited from Guangdong Provincial People’s Hospital (Guangdong Academy of Medical Sciences) affiliated to Southern Medical University, China. Based on clinical and laboratory evaluations, participants were classified into two groups: those with active Mtb patients and healthy controls. The diagnosis of active TB was a positive result for Mtb culture or GeneXpert MTB/RIF assay in designated samples, such as sputum or bronchoalveolar lavage fluid. Healthy controls were required to have no clinical signs or symptoms suggestive of TB, no history of tuberculosis, and negative microbiological results and radiographic imaging. To avoid confounding immune or biochemical alterations, individuals were excluded if they were younger than 18 years of age, had received anti-TB treatment within the past four weeks, or had incomplete clinical or laboratory information. All enrolled participants underwent clinical evaluation to ensure accurate classification. The study was approved by the Medical Ethics Committee of Xuzhou Infectious Disease Hospital (approval number: XCYPJ-2025012001), which was conducted following good clinical practice guidelines. A schematic overview of the study workflow is shown in **Figure 1**.

### 2.2 Routine laboratory tests

Routine laboratory tests were performed using standard procedures for both hematological and biochemical analyses. For hematological tests, ethylenediaminetetraacetic acid (EDTA)-anticoagulated peripheral blood samples were collected, and a complete blood count (CBC) was conducted using the XN-9000 Sysmex analyzer (Sysmex Co., Kobe, Japan) following the manufacturer’s instructions. The parameters assessed included white blood cell count (WBC), red blood cell count (RBC), neutrophil count (NEU), lymphocyte count (LYM), monocyte count (MON), eosinophil count (EOS), basophil count (BAS), red blood cell distribution width coefficient of variation (RDW-CV), standard deviation of red cell distribution width (RDW-SD), mean corpuscular volume (MCV), hematocrit (HCT), hemoglobin (HGB), mean corpuscular hemoglobin (MCH), mean corpuscular hemoglobin concentration (MCHC), platelet count (PLT), Plateletcrit (PCT), mean platelet volume (MPV), platelet distribution width (PDW), platelet larger cell ratio (P-LCR), and platelet large cell count (PLCC). For biochemical analysis, heparin-anticoagulated peripheral blood samples were collected, and the biochemical parameters were measured using the ROCHE COBAS analyzer (Mannheim, Germany) according to the manufacturer’s guidelines. The biochemical markers assessed included total protein (TP), albumin (ALB), globulin (GLOB), total bilirubin (TBIL), direct bilirubin (DBIL), indirect bilirubin (IBIL), creatinine (CREA), uric acid (UA), glucose (GLU), total cholesterol (CHOL), triglycerides (TG), high-density lipoprotein cholesterol (HDL-C), and low-density lipoprotein cholesterol (LDL-C). All analytical procedures were performed under strict quality control measures to ensure the reliability and accuracy of the results. To prepare laboratory indicators for model development, standard preprocessing steps were applied. These included data cleaning, removal of samples with missing or incomplete laboratory information, and harmonization of variable formats across cohorts. A correlation analysis of all laboratory variables was conducted, and highly collinear features with redundant biological information were removed.

### 2.3 Construction of machine learning models

To obtain an effective identification model for diagnosing active Mtb patients and healthy individuals, we compared the performance of six ensemble learning algorithms: Adaptive Boosting (AdaBoost), Bootstrap Aggregating (Bagging), Categorical Boosting (CatBoost), Random Forest (RF), Support Vector Machine (SVM), and eXtreme Gradient Boosting (XGBoost), implemented with the *Scikit-Learn* package (version 0.21.3). The dataset was preprocessed using the *LabelEncoder* function and the *to_categorical* method from *Scikit-Learn* to convert example labels into label-encoded forms. We divided the dataset into 70% for the training cohort and 30% for the validation cohort (internal validation). The training cohort was used for model training and hyperparameter optimization, while the validation cohort was used to assess the model’s performance on unseen data (Xu and Goodacre, 2018). This division ensures that the models are validated on different datasets, reducing the risk of overfitting and enhancing the generalizability of the models (Kernbach and Staartjes, 2021). To further validate the generalizability and robustness of the established models, the models were subsequently evaluated on an independent cohort. During the training of the six machine learning models, we employed the *GridSearchCV* function to train and optimize the hyperparameter combination. This procedure aimed to identify the best-fitting parameters for each model.

### 2.4 Model validation and interpretation

To evaluate the diagnostic performance of machine learning models in distinguishing between active Mtb patients and healthy individuals, quantitative metrics were employed to assess the performance of the machine learning models. Common evaluation metrics such as accuracy, precision, recall, and F1-score were used to evaluate the generalization ability of these models (Yacouby and Axman, 2020). To mitigate the overfitting risk during model training, we implemented 5-fold CV by setting cv = 5 in the *cross_val_score* function, which partitioned the training dataset into five equally sized subsets for iterative validation (Yadav and Shukla, 2016). Additionally, the area under the curve (AUC) value, derived using the *roc_auc_score* function, was incorporated as a performance metric to account for potential class imbalance (Marzban, 2004). 95% confidence intervals (CIs) for AUC were further estimated by bootstrap with 1,000 resamples of the test set, and the percentile method was used to derive the 95% CIs. To visually represent the predictive performance of the best-performing model on the test dataset, we generated a confusion matrix using the *confusion_matrix* function, which displayed the results in a 2*2 matrix format corresponding to the binary classification task (Markoulidakis and Markoulidakis, 2024). Model calibration was evaluated by plotting calibration curves and calculating the Brier score using the calibration_curve and brier_score_loss functions, respectively. Additionally, due to the challenge of correctly interpreting machine learning models, we introduced the SHAP method to rank and visualize the importance of laboratory indicators (Li, 2022). Specifically, *shap.TreeExplainer* was invoked to load the generated model file and the test data. Then, *explainer.shap_values* were computed for features of each sample. The resulting SHAP values were input into *shap.summary_plot* to reflect feature importance and the contribution of each feature to positive and negative predictions for the samples.

### 2.5 External cohort validation

An independent external validation cohort was collected from patients at Guangdong Provincial People’s Hospital (Guangdong Academy of Medical Sciences), China, with inclusion and exclusion criteria consistent with those applied to the discovery cohort. The external validation cohort was used as input for the optimal model, and the same evaluation metrics as the derivation cohorts were used to measure the performance of the model on unknown data. As the purpose of external validation was to assess out-of-sample predictive performance rather than optimize hyperparameters, five-fold cross-validation was not performed on the validation cohort.

### 2.6 Statistical analysis

Descriptive statistics were used to summarize demographic and clinical characteristics of study participants. Continuous variables were presented as medians with ranges, and categorical variables were expressed as counts and percentages. Comparisons between different groups were performed using the Mann-Whitney U test for continuous variables, and the Chi-square test was applied to categorical variables. *P* < 0.05 was considered statistically significant. All statistical analyses were conducted using GraphPad Prism (version 9.3.0) and R programming language (version 4.3.2).

## 3 Results

### 3.1 Characteristics of study participants

A total of 4,234 participants were enrolled in this study, comprising 3829 individuals from Xuzhou Infectious Diseases Hospital (The 7^th^ Hospital of Xuzhou) for model development (the discovery cohort) and 405 individuals from Guangdong Provincial People’s Hospital for external validation. In the discovery cohort, 1,967 patients were diagnosed with active Mtb infection, and 1,862 were healthy individuals. The validation cohort consisted of 205 active Mtb cases and 200 healthy controls. A comparison of baseline demographic characteristics between the active Mtb and healthy groups is presented in **Table 1**. No significant differences in age or sex distribution were observed between the active Mtb and healthy groups in either the discovery cohort or the validation cohort.

**Table 1:** Basic information form of participants in the health group and active Mtb patients, including age and gender.

Cohorts	Discovery cohort		Validation cohort	
	Health (n = 1862)	Active Mtb (n = 1967)	Health (n = 200)	Active Mtb (n = 205)
Median age, year (range)	53 (18-69)	48 (18-98)	49 (18-66)	46 (18-85)
*P*-value	0.247		0.412	
Sex, male,%	1239 (66.54%)	1323 (67.25%)	117 (58.50%)	131 (63.90%)
*P*-value	0.662		0.311	

### 3.2 Performance of routine laboratory indicators in discriminating active Mtb patients from healthy population

To evaluate the discriminatory power of routine hematological and biochemical indicators between active Mtb patients and healthy individuals, comparative analysis and ROC curve assessments were conducted across all variables. As shown in **Table 2**, multiple hematological indicators exhibited statistically significant differences between the two groups. Notably, active Mtb patients had significantly lower values of RBC, LYM, HCT, HGB, RDW-SD, MPV, and P-LCR (P < 0.05), suggesting altered erythrocyte and platelet parameters associated with TB pathology (Iqbal et al., 2015). Conversely, NEU levels were significantly elevated in the active Mtb group (P < 0.05), aligning with studies demonstrating neutrophil-driven inflammation in TB (Kerkhoff et al., 2013). Among biochemical indicators, ALB, TP, CHOL, HDL-C, LDL-C, and GLOB were significantly lower or altered in active Mtb patients compared to healthy individuals (P < 0.05), reflecting systemic inflammatory and nutritional disturbances (Chidambaram et al., 2021). ALT, TBIL, DBIL, CREA, and UA levels showed no significant differences (P > 0.05), indicating limited diagnostic value for TB differentiation.

**Table 2:** Comparison of hematological and biochemical parameters between active Mtb patients and healthy individuals.

Indicators	Health(n=1862)	Active Mtb(n=1967)	*P*-value
Complete blood count, median (range)			
WBC (×10^9^/L)	6.14 (2.69, 22.26)	5.80 (1.40, 23.37)	0.878
RBC (×10¹²/L)	4.64 (2.70, 6.70)	4.36 (1.67, 6.48)	0.000*
NEU (×10^9^/L)	3.42 (1.03, 19.52)	3.83 (1.65, 20.23)	0.002*
LYM (×10^9^/L)	2.05 (0.72, 5.46)	1.36 (0.07, 4.51)	0.000*
MON (×10^9^/L)	0.35 (0.08, 3.17)	0.38 (0.01, 2.63)	0.174
EOS (×10^9^/L)	0.11 (0.00, 1.09)	0.10 (0.00, 3.78)	0.289
BAS (×10^9^/L)	0.01 (0.00, 0.14)	0.02 (0.00, 0.92)	0.208
RDW-CV (%)	13.10 (10.50, 20.70)	13.00 (10.50, 22.80)	0.785
RDW-SD (fL)	45.90 (34.10, 90.90)	41.80 (28.90, 82.50)	0.000*
MCV (fL)	90.90 (59.90, 122.40)	89.30 (48.50, 119.20)	0.000*
HCT (%)	42.60 (26.20, 76.70)	38.40 (15.70, 58.70)	0.000*
HGB (g/L)	136.00 (74.00, 191.00)	128.00 (44.00, 195.00)	0.000*
MCH (pg)	29.50 (15.00, 48.10)	29.70 (17.10, 39.20)	0.946
MCHC (g/L)	330.00 (196.00, 445.00)	332.00 (234.00, 398.00)	0.019*
PLT (×10^9^/L)	231.00 (47.00, 623.00)	236.00 (23.00, 1078.00)	0.054
PCT (%)	0.24 (196.00, 445.00)	332.00 (234.00, 398.00)	0.000*
MPV(fL)	10.30 (7.60, 17.40)	8.30 (5.40, 15.10)	0.000*
PDW (%)	16.30 (11.00, 35.3)	15.70 (14.40, 17.60)	0.000*
P-LCR (%)	23.20 (6.60, 93.60)	18.20 (2.00, 84.00)	0.010*
Biochemical indicators, median (range)			
ALT (U/L)	17.00 (1.00, 400.00)	15.00 (2.00, 765.00)	0.268
AST (U/L)	19.00 (2.00, 217.00)	21.00 (6.00, 887.00)	0.014*
TP (g/L)	73.48 (43.28, 101.80)	69.40 (30.50, 85.20)	0.000*
ALB (g/L)	45.99 (22.80, 56.80)	38.65 (0.80, 52.90)	0.000*
GLOB (g/L)	27.47 (11.70, 52.70)	30.80 (14.90, 63.50)	0.000*
TBIL (μmol/L)	11.62 (2.57, 353.60)	9.10 (1.80, 460.00)	0.521
DBIL (μmol/L)	3.40 (0.00, 284.80)	3.10 (0.00, 294.30)	0.432
IBIL (μmol/L)	8.26 (0.78, 85.80)	5.85 (0.00, 165.70)	0.008*
CREA (μmol/L)	66.00 (30.00, 539.00)	61.10 (14.80, 838.40)	0.323
UA (μmol/L)	309.00 (72.00, 1168.60)	283.35 (11.30, 1103.80)	0.303
GLU (mmol/L)	5.18 (3.43, 23.67)	4.95 (0.02, 29.71)	0.661
CHOL (mmol/L)	4.77 (1.87, 11.53)	3.84 (0.64, 11.70)	0.000*
TG (mmol/L)	1.01 (0.24, 18.32)	0.90 (0.01, 10.51)	0.010*
HDL-C (mmol/L)	1.31 (0.59, 3.40)	0.93 (0.01, 2.82)	0.000*
LDL-C (mmol/L)	3.07 (0.68, 6.55)	2.09 (0.22, 9.04)	0.000*

*footnote: bold values indicate statistical significance.

**Figure 2:**
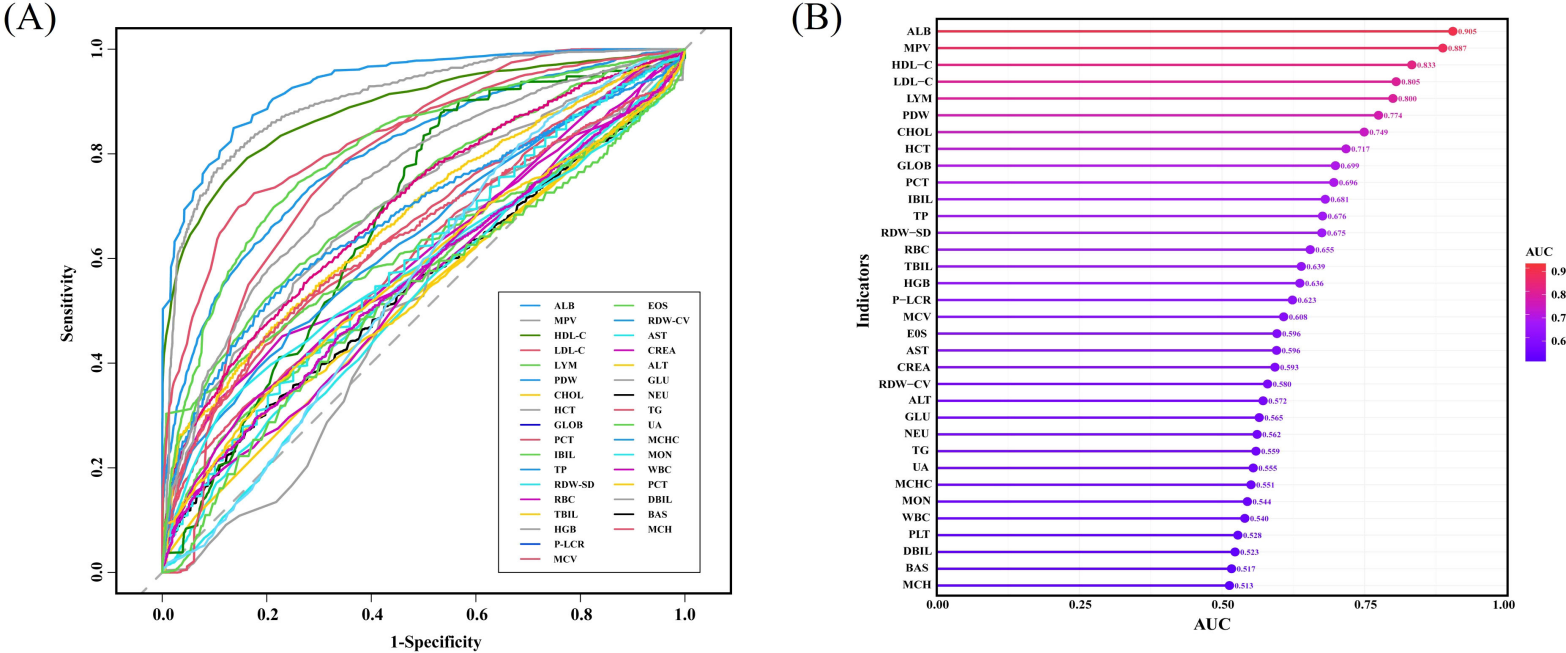
Performance of individual indicators from routine laboratory tests in differentiating active MTB patients from healthy individuals. **(A)** ROC curves showing the diagnostic performance of individual indicators from routine laboratory tests in discriminating between active Mtb and healthy individuals. **(B)** Cleveland dot plot showing the AUC of various indicators in discriminating active MTB patients from healthy individuals.

To further quantify the diagnostic capacity of these indicators, ROC curve analysis was performed (**Figure 2A**), and the AUC for each marker was calculated and ranked (**Figure 2B**). The top-performing indicators were ALB, MPV, HDL-C, LDL-C, and LYM, each achieving an AUC greater than 0.8, indicating strong individual predictive power in differentiating active Mtb from healthy controls.

### 3.3 Machine learning model comparison and external validation

**Figure 3:**
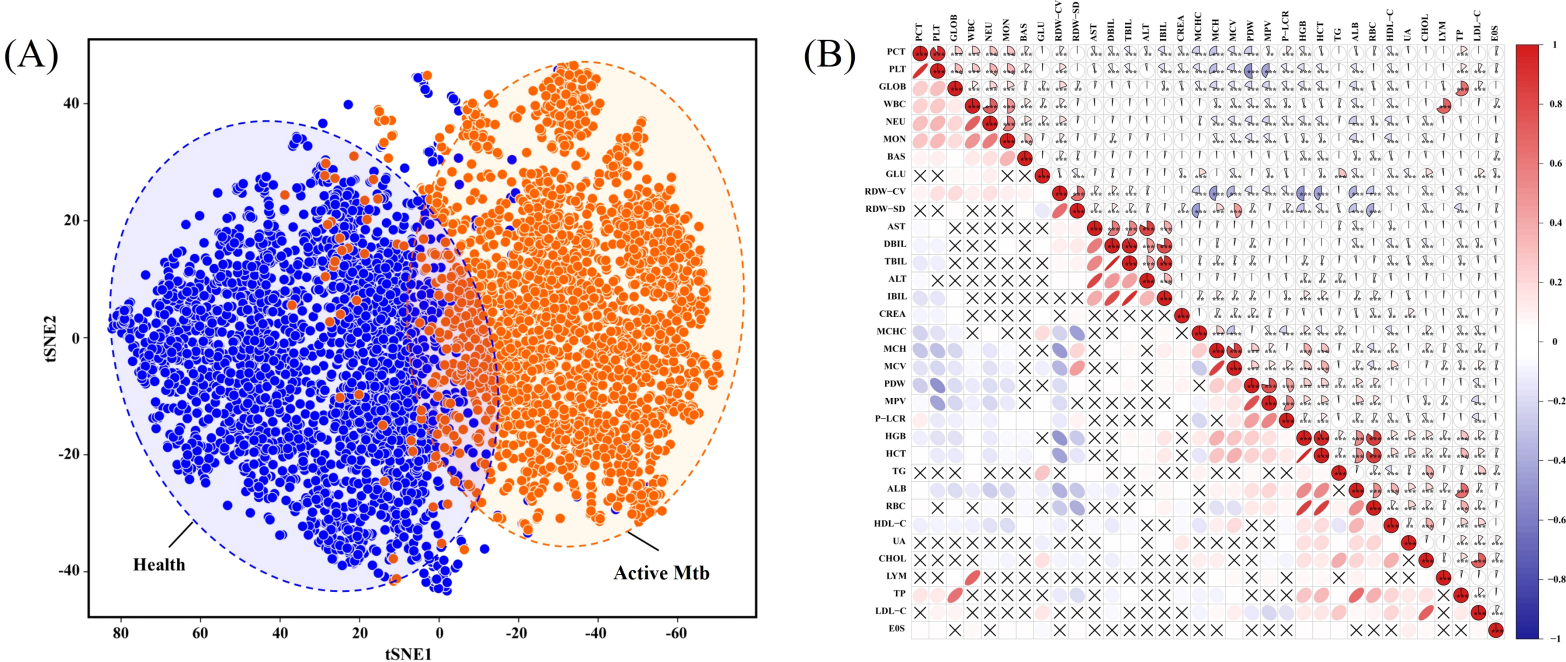
Application of routine laboratory indicators in differentiating active MTB patients from healthy individuals. **(A)** t-SNE plot illustrating the two-dimensional distribution of routine laboratory indicators. **(B)** The correlation plot shows the correlations among the various indicators in the active Mtb group.

To evaluate the feasibility of integrating multiple routine laboratory indicators for the classification of active Mtb infection, dimensionality reduction and correlation analysis were first performed to investigate the underlying structure and relationships among the selected features. As shown in **Figure 3A**, the result of t-SNE demonstrates a clear separation between active Mtb patients and healthy individuals based on the full set of hematological and biochemical features. Despite some degree of overlap at the boundary regions, the clustering pattern indicates that the combined feature space can effectively differentiate the two groups in a non-linear, high-dimensional context. Furthermore, Pearson Correlation Analysis among laboratory indicators in the active Mtb group identified distinct patterns of association (**Figure 3B**). For instance, NEU exhibited a strong positive correlation with WBC, reflecting coordinated elevation in systemic inflammation (Hu et al., 2014). Similarly, PLT exhibited a high correlation with PCT, indicating reactive thrombocytosis potentially induced by systemic inflammation (Peng et al., 2017). In contrast, RDW-SD was negatively correlated with HGB and HCT, suggesting that worsening anisocytosis may be associated with anemia of chronic disease in active Mtb (Ashenafi et al., 2022). These complex multivariate interactions highlight the limitations of traditional univariate approaches and underscore the necessity of machine learning models, which can capture non-linear relationships and automatically adjust feature weights to optimize classification performance.

To further assess the diagnostic utility of routine laboratory indicators in distinguishing active Mtb infection, we constructed and evaluated six supervised machine learning models, including AdaBoost, Bagging, CatBoost, RF, SVM, and XGBoost. Their performance was assessed using multiple evaluation metrics, including accuracy, precision, sensitivity (recall), F1-score, and 5-fold cross-validation (**Table 3**). Among all models, XGBoost achieved the best overall performance, with an accuracy of 97.49%. These results indicate that XGBoost not only correctly identifies a high proportion of active Mtb cases (sensitivity), but also maintains strong ability to exclude healthy individuals (specificity), making it a balanced and reliable classifier. Its robustness was further confirmed by the highest 5-fold cross-validation accuracy (97.20%), suggesting minimal overfitting. The superior performance of XGBoost may be attributed to its ability to capture complex nonlinear relationships and interactions among routine laboratory features through gradient boosting and regularization (Dong et al., 2022). It provides built-in methods for estimating feature importance, which facilitates model interpretability and can help identify key clinical variables in complex, high-dimensional datasets (Takefuji). The SVM model performed comparably well, yielding a balanced accuracy of 97.15%, and also demonstrated strong generalization capability. Bagging and CatBoost followed closely, with accuracy ranging from 95.86% to 96.26%, indicating solid but slightly less consistent performance across metrics. In contrast, RF exhibited the lowest predictive performance among the ensemble models, with an overall accuracy of 94.14%. Due to its superior performance in the internal evaluation, the XGBoost model was selected for external validation using an independent cohort from Guangdong Provincial People’s Hospital. In this validation set, the model maintained strong predictive ability, achieving an accuracy of 93.67%, a sensitivity of 91.56%, and a specificity of 91.13%.

**Table 3:** Performance comparison of six supervised machine learning algorithms for prediction of routine indicators in active Mtb infection diagnosis.

Algorithm	Accuracy	Prediction	Sensitivity	Specificity	F1-score	5Fold-CV
XGBoost	97.49%	97.49%	97.56%	97.32%	97.52%	97.20%
SVM	97.15%	97.15%	97.06%	97.08%	97.10%	96.89%
Bagging	96.26%	96.26%	96.26%	96.13%	96.26%	95.87%
CatBoost	95.86%	95.86%	95.86%	95.65%	95.86%	95.28%
AdaBoost	94.46%	94.46%	94.49%	94.26%	94.47%	94.35%
RF	92.24%	92.24%	92.13%	92.18%	92.18%	92.04%
External cohorts (XGBoost)						
GDPH^*^	93.67%	93.67%	91.56%	91.13%	92.60%	N/A

^*^GDPH, Guangdong Provincial People’s Hospital.

In addition, we conducted subgroup analyses based on sex and age in the external validation cohort. For the sex-based subgroups, the XGBoost model showed comparable performance in both male and female groups, with accuracy values of 94.35% and 93.23%, respectively. Both groups exhibited high sensitivity and specificity, indicating the model’s consistent performance across gender. For age-based subgroups, the model also demonstrated strong performance. In the 18–40 group, the accuracy was 93.77%, while in the 40–60 group, it was slightly higher at 93.86%. For the ≥60 age group, the accuracy decreased to 91.55%, but the model still maintained a strong performance in this subgroup with sensitivity and specificity remaining relatively high (**Supplementary Table S1**). These findings underscore the model’s ability to generalize across different clinical settings, patient demographics, and laboratory conditions.

### 3.4 Model validation and interpretation

**Figure 4:**
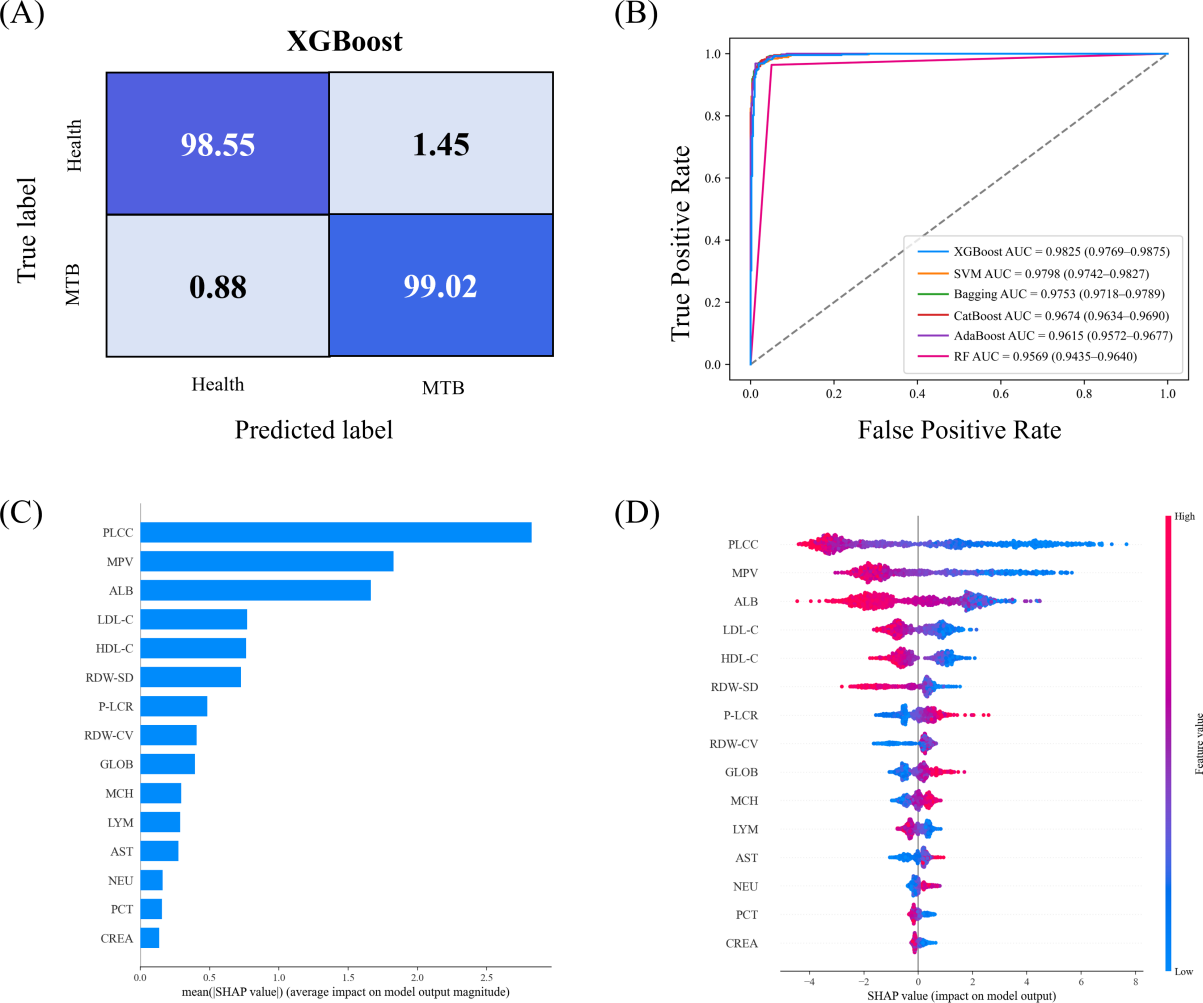
Diagnostic performance and feature importance analysis of the machine learning models. **(A)** Confusion matrix for the XGBoost model in discriminating between active MTB and healthy individuals. **(B)** ROC curves comparing the diagnostic performance of various machine learning models. **(C)** Mean SHAP values showing the importance of individual features in the XGBoost model. **(D)** SHAP summary plot illustrating the impact of individual features on the XGBoost model’s output.

To further verify the diagnostic utility of the XGBoost model and enhance its clinical interpretability, we performed a comprehensive evaluation of its classification performance and feature attribution behavior. As shown in **Figure 4A**, the confusion matrix revealed that the XGBoost model achieved high classification accuracy in distinguishing active Mtb patients from healthy individuals. The true positive rate (correctly identified Mtb cases) was 98.02%, and the true negative rate (correctly identified healthy individuals) was 97.65%, indicating excellent discrimination capability. ROC curves were used to compare the diagnostic performance of all machine learning models (**Figure 4B**). The AUC of the XGBoost model reached 0.9825 (95% CI 0.9769–0.9875), the highest among all models evaluated. In addition to discriminati on performance, we also assessed the calibration of the XGBoost model to evaluate the agreement between predicted probabilities and actual event rates (**Supplementary Figure S1**). The calibration curve showed generally good agreement between predicted probabilities and observed event rates, with a Brier score of 0.047, indicating acceptable overall calibration, although some fluctuations were seen in intermediate risk groups. Together, these results confirm that the XGBoost model exhibits both strong discriminatory power and reliable probability estimation, supporting its potential applicability in clinical decision-making.

To enhance model interpretability and facilitate clinical understanding of the key drivers of prediction, we employed SHAP to quantify feature contributions at both global and individual levels (Prendin et al., 2023). The mean SHAP value (**Figures 4C, D**) further illustrates the contribution and directionality of individual features, offering refined interpretability at both global and instance levels (**Supplementary Table S2**). Among the top-ranked features, ALB demonstrated the strongest negative association with the model’s prediction of active Mtb infection. Specifically, lower ALB values contributed markedly to positive model outputs (i.e., classification as active Mtb), consistent with the well-established role of hypoalbuminemia as a biomarker of systemic inflammation, malnutrition, and disease severity in TB.

MPV was also identified as a major contributor. Lower MPV values were strongly associated with the active Mtb class, reflecting possible platelet consumption and microvascular activation commonly observed during chronic infectious processes (Korniluk et al., 2019). Both LDL-C and HDL-C exhibited strong negative SHAP contributions, where reduced levels were linked to increased model probability of active Mtb. These findings are in line with reports of lipid metabolism remodeling during active Mtb infection, possibly reflecting host-pathogen competition for lipid substrates and altered hepatic synthesis under inflammatory stress (Akpovi et al., 2013). Other hematological features, including RDW-SD and MCH, showed meaningful contributions as well. Anemia and malnutrition are common comorbidities in patients with active Mtb, often resulting from chronic inflammation, reduced dietary intake, and impaired nutrient absorption (Chhabra et al., 2021). In this context, elevated RDW-SD values in active Mtb patients may reflect increased variability in red blood cell size, associated with nutritional deficiencies such as iron, folate, or vitamin (Garg).

Notably, LYM and PCT also contributed to the model’s decision boundary, albeit to a lesser extent. Lymphopenia is frequently observed in advanced or disseminated TB and may indicate underlying immune dysregulation (Luo et al., 2021). PCT, reflecting total platelet mass, may complement MPV in capturing platelet activation and production dynamics in response to infection. Together, the SHAP interpretability framework confirms that the XGBoost model not only achieves high predictive performance but also relies on clinically relevant features with mechanistic plausibility. This enhances the model’s credibility and potential for translation into practical diagnostic workflows.

## 4 Discussion

TB remains one of the most prevalent and deadly infectious diseases worldwide, particularly affecting low- and middle-income countries (Mohajan, 2014). With an estimated 10 million new cases each year, TB continues to impose a heavy burden on global health, particularly affecting vulnerable groups such as those living with HIV/AIDS, the malnourished or those with limited access to healthcare (Kwan and Ernst, 2011; Foo et al., 2022). Rapid and accurate identification of active Mtb infection is essential for curbing transmission, initiating timely treatment, and improving clinical outcomes. Conventional diagnostic methods, such as sputum smear microscopy, mycobacterial culture, and chest radiography, are often limited by low sensitivity, prolonged turnaround time, or poor specificity (Ryu, 2015). These techniques are often time-consuming, technically demanding, or cost-prohibitive, especially in resource-limited settings, which limits their widespread implementation and delays diagnosis and treatment (Parsons et al., 2011). Therefore, there is an urgent need to develop alternative diagnostic approaches that are accurate, low-cost, minimally invasive, and amenable to broad implementation across diverse clinical environments.

In recent years, increasing evidence has highlighted the potential of combining routine laboratory indicators with ML algorithms to enhance diagnostic performance for various diseases (Tang et al., 2021). Numerous studies have demonstrated the practical utility of hematological and biochemical features within data-driven diagnostic frameworks (Silva, 2024; Yang et al., 2024). For instance, Alves et al. (2021) applied a random forest model to routine blood test data for COVID-19 screening, achieving an AUC of 0.86 and demonstrating good overall accuracy and interpretability using decision tree–based explanation tools. Similarly, Xu et al. (2025) developed a gradient boosting model combining tumor markers and routine blood indices to detect colorectal cancer, achieving an AUC above 0.90 in both internal and external validation cohorts. These studies highlight the scalability, accessibility, and clinical relevance of combining widely available blood data with machine learning techniques, especially in low-resource settings that lack advanced diagnostic infrastructure.

In this context, we developed a diagnostic framework that combines routine hematological and biochemical indicators from peripheral venous blood with supervised machine learning algorithms to identify active Mtb. To fully exploit the diagnostic potential of these routine blood indicators, we employed a multi-step analytical pipeline. Initially, univariate statistical comparisons and ROC curve analyses were performed to evaluate the discriminative power of individual features. The AUC values for several metrics including ALB, MPV, HDL-C, LDL-C, and LYM were all greater than 0.80, underscoring their potential as candidate biomarkers of active Mtb disease. To further assess group separation in high-dimensional space, we utilized t-SNE, which revealed clear clustering between TB cases and healthy controls.

Based on these findings, we constructed and evaluated six supervised machine learning models. Among them, the XGBoost algorithm demonstrated the most robust and reliable performance, achieving an accuracy of 97.49%, a sensitivity of 97.56%, and a specificity of 97.42% in the internal cohort. External validation using an independent dataset further confirmed its strong generalizability, with an accuracy of 93.67%. Several methodological characteristics likely contributed to the superior performance of XGBoost. As a gradient boosting framework, XGBoost effectively captures the complex, non-linear, and high-order interactions among hematological and biochemical features that characterize active tuberculosis. In addition, the incorporation of both L1 and L2 regularization within its objective function reduces overfitting and ensures stable learning from high-dimensional and heterogeneous clinical data. Its inherent robustness to missing values and measurement variability further enhances its suitability for real-world laboratory datasets. Compared to traditional diagnostic methods, this data-driven framework presents a cost-effective and scalable that integrates computational efficiency with clinical accessibility. Beyond high predictive accuracy, an important advantage of the XGBoost model is that its SHAP-derived feature contributions align closely with the known pathophysiology of active Mtb infection, thereby reinforcing the biological plausibility of our findings. ALB emerged as the strongest predictor, a result consistent with the profound systemic inflammation and catabolic state characteristic of tuberculosis; pro-inflammatory cytokines suppress hepatic albumin synthesis, while chronic infection increases metabolic consumption, jointly leading to hypoalbuminemia (Fayed et al., 2018). Platelet-related indices such as MPV and PCT were also highly influential, reflecting inflammation-driven platelet activation, consumption, and a shift toward smaller platelet populations (Verma et al., 2025). Additionally, reductions in HDL-C and LDL-C likely reflect Mtb-induced remodeling of host lipid metabolism, as the pathogen relies heavily on host lipids for persistence, while inflammatory signaling further suppresses hepatic lipoprotein synthesis (Baluku et al., 2024). Hematologic markers including RDW-SD, MCH, and LYM also contributed meaningfully to model predictions. Elevated RDW-SD indicates impaired erythropoiesis and increased erythrocyte size variability, common consequences of chronic inflammation, nutritional deficiencies, and anemia associated with tuberculosis, while lymphopenia reflects T cell depletion, apoptosis, or peripheral redistribution, all of which are associated with disease severity and bacterial burden (Selvavinayagam et al., 2024). These associations support the biological plausibility and clinical relevance of our model’s predictions.

Furthermore, evidence synthesized by Zorina et al. indicates that persistent viral and bacterial infections can trigger long-term systemic inflammation, sustained cytokine release, oxidative stress, and metabolic dysregulation (Zorina et al., 2023). All these effects may extend beyond the primary site of infection and lead to neuroinflammation and tissue degeneration. These mechanisms are highly relevant to the host responses observed in active Mtb infection. The chronic inflammatory environment unique to tuberculosis aligns with prolonged immune activation, where cytokine network dysregulation, immune cell exhaustion, and metabolic reprogramming shape systemic physiological changes. Such processes provide additional context for the SHAP-identified predictors in our model, including hypoalbuminemia, dyslipidemia, altered platelet indices, and lymphopenia, which reflect not only TB-specific immune responses but also fundamental host-pathogen interaction patterns shared across chronic infectious diseases.

Beyond diagnostic performance and biological interpretability, the practical implementation of this model in real-world clinical workflows is an important consideration. The XGBoost model requires relatively modest computational resources once trained, and the prediction process can be executed on standard hospital computers or server environments without the need for dedicated hardware. This allows the model to be embedded into existing laboratory workflows with minimal technical burden. The structure of the input data, which consists of routine hematological and biochemical indicators, is already compatible with laboratory information systems used in most clinical settings. The model can therefore be integrated into these systems through automated data extraction and real-time result generation, enabling seamless support for diagnostic decision making. All data used in this study were fully anonymized, and any clinical deployment would comply with institutional and national data protection regulations, with safeguards such as secure data transmission and controlled access. With these requirements met, the model could serve as a practical and low-cost decision support tool that facilitates early identification of active Mtb infection, particularly in high-burden or resource-limited settings.

Despite the promising results, this study has several limitations. First, although external validation was performed, the independent cohort was drawn from a single center, which may limit generalizability. We further acknowledge that hospital-based controls may not fully represent individuals encountered in community-wide screening programs, and this mismatch may introduce a degree of spectrum bias. Hospital-attending populations often differ from community individuals in terms of comorbidities, healthcare-seeking behaviors, and baseline laboratory characteristics, whereas community participants may be asymptomatic or present with subclinical disease. While such bias cannot be entirely avoided in retrospective hospital-based studies, the inclusion of an external validation cohort helps partially mitigate this concern by assessing model performance in a population distinct from the derivation set. Potential confounding factors, including comorbidities, concurrent medications, and nutritional status, may also influence routine laboratory indicators independent of tuberculosis. Although strict inclusion criteria were applied to minimize these effects, residual confounding remains possible due to incomplete information on these variables in retrospective datasets. Future prospective studies with comprehensive clinical documentation and community-based sampling, particularly those including individuals with latent or subclinical tuberculosis, will be essential for more rigorous assessment of model robustness across diverse populations. Second, our model relied solely on routine laboratory parameters. Incorporating other clinical data, such as symptoms, imaging findings, or comorbidities, can further enrich the feature space and enhance the robustness of the diagnosis. These approaches integrate transcriptomic, proteomic, and epigenomic information within spatial contexts, offering a deeper understanding of how microenvironmental cues shape disease initiation and progression. As spatial and multimodal omics technologies continue to mature, future studies that combine routine laboratory indicators with spatial or multi-omic data may further enhance the mechanistic interpretability and biological relevance of tuberculosis prediction models.

In conclusion, our findings demonstrate that routine blood-derived indicators, when integrated with interpretable machine learning models, can accurately and reliably identify active Mtb. Routine hematological and biochemical tests are inexpensive, widely available, and rapidly obtained in most healthcare settings, including primary care facilities. Owing to these advantages, the proposed model could serve as an initial risk stratification tool to identify individuals who would benefit most from confirmatory testing. Patients predicted to be at higher risk could be prioritized for GeneXpert testing, radiographic evaluation, or microbiological assays, which would improve diagnostic efficiency and reduce unnecessary use of more resource-intensive procedures. In settings with limited access to advanced diagnostics, the model may also function as a preliminary screening tool to support clinical decision making. This complementary, stepwise approach aligns well with real-world clinical workflows and has the potential to enhance early detection, reduce diagnostic delays, and optimize resource allocation. Together, these strengths indicate that this approach offers a practical, scalable, and cost-effective diagnostic aid that supports tuberculosis control efforts, particularly in high-burden and resource-constrained environments.

## Data Availability

The dataset will be available upon request. Requests to access these datasets should be directed to Liang Wang, healthscience@foxmail.com.
