# Supplementary material for: Validation and interpretation of machine-learning models for rapid identification of active tuberculosis infection using routine laboratory indicators
Source: Front Cell Infect Microbiol. 2025 Dec 18;15:1718614. doi: 10.3389/fcimb.2025.1718614 (PMC12756366; doi:10.3389/fcimb.2025.1718614)
Supplement: Supplementary file 1 [file Table1.docx]

**Supplementary Figure S1** Calibration plot of the XGBoost model. The blue line represents the observed event rate within deciles of predicted probability, and the orange dashed line indicates perfect calibration (predicted probability equal to observed frequency).
