# Supplementary material for: Validation and interpretation of machine-learning models for rapid identification of active tuberculosis infection using routine laboratory indicators
Source: Front Cell Infect Microbiol. 2025 Dec 18;15:1718614. doi: 10.3389/fcimb.2025.1718614 (PMC12756366; doi:10.3389/fcimb.2025.1718614)
Supplement: Supplementary file 2 [file Table2.docx]

| **Feature** | **XGBoost** | **Accuracy** | **Prediction** | **Sensitivity** | **Specificity** | **F1-score** |
| --- | --- | --- | --- | --- | --- | --- |
| Sex | Male (n=248) | 94.35% | 94.32% | 93.28% | 93.41% | 93.80% |
|  | Female (n=157) | 93.23% | 93.20% | 92.15% | 92.18% | 92.67% |
| Age, year | 18-40 (n=132) | 93.77% | 93.54% | 92.41% | 92.52% | 92.97% |
|  | 40-60 (n=202) | 93.86% | 93.74% | 92.50% | 92.63% | 93.12% |
|  | ≥60 (n=71) | 91.55% | 91.48% | 90.43% | 90.67% | 90.95% |

**Supplementary Table S1** Diagnostic performance of the XGBoost model in subgroups of the external validation cohort stratified by sex and age.
