# Supplementary material for: Validation and interpretation of machine-learning models for rapid identification of active tuberculosis infection using routine laboratory indicators
Source: Front Cell Infect Microbiol. 2025 Dec 18;15:1718614. doi: 10.3389/fcimb.2025.1718614 (PMC12756366; doi:10.3389/fcimb.2025.1718614)
Supplement: Supplementary file 3 [file Table3.docx]

**Supplementary Table S2** Laboratory features included in the XGBoost model and their mean SHAP importance values.

| **Feature** | **Mean_SHAP_Value** |
| --- | --- |
| ALB | 3.50988 |
| MPV | 2.9404488 |
| LDL-C | 2.4903293 |
| HDL-C | 2.0257163 |
| MCH | 1.1774217 |
| RDW-SD | 1.1405752 |
| LYM | 0.8896867 |
| PCT | 0.8533466 |
| HGB | 0.7837917 |
| GLOB | 0.7833046 |
| CHOL | 0.75732064 |
| AST | 0.6922508 |
| ALT | 0.67122346 |
| RDW-CV | 0.5893227 |
| GLU | 0.537756 |
| MCV | 0.4511316 |
| HCT | 0.38450438 |
| P-LCR | 0.37484714 |
| PDW | 0.35144717 |
| MCHC | 0.3059459 |
| TG | 0.30033073 |
| DBIL | 0.29253784 |
| TBIL | 0.29239768 |
| TP | 0.25987893 |
| CREA | 0.2241732 |
| RBC | 0.21620515 |
| MON | 0.2115968 |
| E0S | 0.18863831 |
| IBIL | 0.18500401 |
| NEU | 0.18460266 |
| PLT | 0.14861463 |
| UA | 0.11372783 |
| WBC | 0.0854977 |
| BAS | 0.0533469 |
